# Microbial comparative pan-genomics using binomial mixture models

**DOI:** 10.1186/1471-2164-10-385

**Published:** 2009-08-19

**Authors:** Lars Snipen, Trygve Almøy, David W Ussery

**Affiliations:** 1 Biostatistics, Department of Chemistry, Biotechnology and Food Sciences, Norwegian University of Life Sciences, Ås, Norway; 2 Centre for Biological Sequence Analysis, Technical University of Denmark, Lyngby, Denmark

## Abstract

**Background:**

The size of the core- and pan-genome of bacterial species is a topic of increasing interest due to the growing number of sequenced prokaryote genomes, many from the same species. Attempts to estimate these quantities have been made, using regression methods or mixture models. We extend the latter approach by using statistical ideas developed for capture-recapture problems in ecology and epidemiology.

**Results:**

We estimate core- and pan-genome sizes for 16 different bacterial species. The results reveal a complex dependency structure for most species, manifested as heterogeneous detection probabilities. Estimated pan-genome sizes range from small (around 2600 gene families) in *Buchnera aphidicola *to large (around 43000 gene families) in *Escherichia coli*. Results for *Echerichia coli *show that as more data become available, a larger diversity is estimated, indicating an extensive pool of rarely occurring genes in the population.

**Conclusion:**

Analyzing pan-genomics data with binomial mixture models is a way to handle dependencies between genomes, which we find is always present. A bottleneck in the estimation procedure is the annotation of rarely occurring genes.

## Background

One of the consequences of the explosion in numbers of fully sequenced and annotated microbial genomes is that we are now facing the challenges of comparative pan-genomics [1]. The microbial pan-genome, as defined by [2], is the number of essentially different genes found within a population at a specified taxonomic level, usually within a species, though this can be extended to higher levels, such as genus. As multiple genomes of the same species are sequenced, one can construct the pan-genome, and begin to compare pan-genomes from different species.

Having a set of fully sequenced and annotated genomes from several strains within a species, one is interested in two sets of genes. The first is the set of core genes, *i.e*. the genes found in every strain within a species. The size and content of the core genome is interesting for characterizing the genomic essence of the species. The other set is the pan-genome, which is the total number of different genes found in all strains within the species. The size of this pan-genome, relative to the number of genes found in a typical strain, is an indicator of the plasticity of the species, and could be reflective of its potential for adaptation in a diverse environment.

The true core- and pan-genome sizes, here denoted *γ *and *η *respectively, will most likely remain unknown for any species, since it is impossible to sequence and annotate all existing strains. Thus, we have to rely on estimates based on existing data. The problem of estimating the size of the core- and pan-genome was first approached by [2]. They used an exponential function to explain the number of new genes introduced by each new sequenced genome, and by extrapolating this they came up with some estimates of the pan-genome size. The core-genome size was also estimated in a similar way. Modified versions of this approach have later been used by others. For example the number of new *Escherichia coli *genes contributed by each additional genome sequenced was first estimated to be rather large – 440 genes by [3]. More recent estimates, based on 17 different isolates from a wide variety of strains, brought the number of expected novel genes per new genome to be around 300, with approximately 13,000 genes estimated to be in the total *E. coli *pan-genome [4]. Based on comparison of 32 *E. coli *genome sequences, we have previously estimated the number to be around 80 novel genes per genome, with a pan-genome size of just under 10,000 genes [5].

One of the implications of early pan-genome estimates is that some bacterial species might have an "infinite" pan-genome [2,6]. This is a dramatic statement, especially since it can be largely due to a bias from their use of an exponential model, which inherently assumes the pan-genome can be divided into two groups: The core-genes always present in all genomes, and the dispensable genes, equally likely to occur in any genome. The latter part of this assumption is often far from reality, which we will show in this paper. This was also recognized by [7], who was the first to introduce a mixture model to estimate the core- and pan-genome size. Unfortunately, they also imposed some rather heavy restrictions in their model, making their pan-genome estimates biased towards larger values.

We will, however, extend the good idea of [7] in this paper, and by avoiding their heavy restrictions hopefully come up with more realistic estimates of core- and pan-genome sizes.

## Results

### Algorithm

#### Gene families

For a given species *G *different genomes have been sequenced and annotated. The first step in any pan-genome analysis is to come up with a list of gene families in the current sample. A deeper analysis of this problem is not the focus of this paper, and we have at this stage taken the approach used by [7] and [5]. First an all-against-all BLASTing (blastp) is performed, and only alignments with at least 50% identity along at least 50% of both sequences are considered. Two sequences belong to the same gene family if both their reciprocal alignments fulfill the 50-50-cutoff rule. The results of this procedure is typically stored in a *pan-matrix ****M ***= {*m*_*ij*_} where each row corresponds to a gene family and each column to a genome. If gene family *i *has at least one member in genome *j *then *m*_*ij *_= 1, else *m*_*ij *_= 0.

#### Mixture model

The pan-genome size, *η*, is the number of gene families found in all strains, also including the gene families not yet observed in the *G *genomes sequenced so far. Summing row *i *in ***M ***we get the number of genomes in which gene family *i *has been observed. Tabulating all these row-sums gives us the number of gene-families observed in 1,..., *G *genomes, which we denote *y*_1_,...*y*_*G*_. The sample pan-genome size is , while *y*_*G *_is usually listed as the sample core-genome size. The true pan-genome size also includes *y*_0_, the number of gene families observed in zero genomes so far. Hence *η *= *n *+ *y*_0 _and estimating *η *is equivalent to predicting *y*_0_.

In order to predict *y*_0 _we need a model that relates *y*_0 _to *y*_1_,..., *y*_*G*_. Consider ***y ***= (*y*_0_, *y*_1_,..., *y*_*G*_). Since the total sum of gene families, *η*, is constant ***y ***is a multiniomial vector if we assume independence between gene families, *i.e*. ***y ***~ Mult(***θ***, *η*). The multinomial probabilities ***θ ***= (*θ*_0_,..., *θ*_*G*_) are the probabilities of a gene family to be detected in 0,..., *G *genomes, respectively. The expected value of *y*_0 _is *E*(*y*_0_) = *ηθ*_0 _due to the multinomial model. Also, a similar argument leads to *E*(*n*) = *η*(1 - *θ*_0_). Combined they lead to(1)

**Figure 1:**
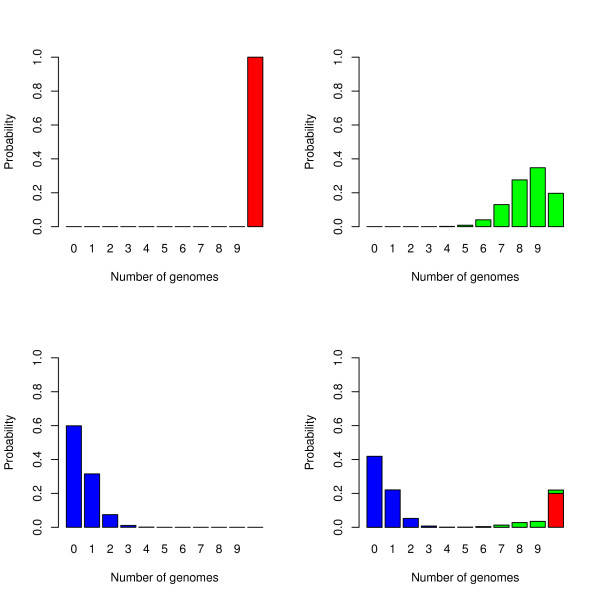
**Mixture model example**. An illustration of a three component binomial mixture model when *G *= 10. The upper left panel shows the binomial probability mass function (PMF, red) for the detection probability *ρ*_1 _= 1.0, i.e. the core component. In the upper right panel a second component has a binomial PMF (green) where *ρ*_2 _= 0.85, and in the lower left panel a third component (blue) has *ρ*_3 _= 0.05. The lower right panel shows their combination into 11 multinomial probabilities, using mixing proportions *π*_1 _= 0.2, *π*_2 _= 0.1 and *π*_3 _= 0.7.

Using *n *as an estimate of *E*(*n*) we can predict *y*_0 _if we can estimate *θ*_0_. This estimate can be found by assuming some degree of smoothness across the multinomial probabilities. One way of obtaining this is by using a binomial mixture model. This means we assume(2)

where *π*_*k *_is the *mixing proportion *and(3)

is a binomial probability mass function with *detection probability ρ*_*k*_. Thus, the multinomial probabilities are expressed as a combination of *K *binomial probability mass functions (PMF). The shape and location of these binomial PMFs will determine how *θ*_*g *_are related to each other, and more specifically how *θ*_0 _relates to *θ*_*g*_, *g *= 1,..., *G*. Figure 1 illustrates this idea for a three component model, i.e. we use the combination of three binomial PMFs to describe the 11 multinomial probabilities. Component *k *in this mixture model may be interpreted as a class of gene families with probability *ρ*_*k *_of being detected (probability of "success") in a genome. If *ρ*_*k *_is low, these genes are typically rarely observed in the sequenced genomes, and vice versa. A binomial mixture like this was also used by [7].

It is natural to reserve one of the mixture components for the class of core genes. Core genes are special, since these genes should always be present in all genomes, and it is natural to assign them detection probability 1.0, as was also done by [7]. We define the first component as the core component, hence *ρ*_1 _= 1.0.

#### Estimation

The parameters of the binomial mixture model cannot be estimated directly from ***y***, again because *y*_0 _is missing. This led [7] to impose some heavy restrictions on their model, which is not necessary. A commonly used approach for such models is to estimate parameters maximizing the zero-truncated log-likelihood [8].

Considering a fixed *n *the vector ***y***_+ _= (*y*_1_,..., *y*_*G*_) is also a multinomial, with probability *θ*_*g*_*/*(1 - *θ*_0_) for element *g *= 1,..., *G*. Thus, the zero-truncated log-likelihood is(4)

where *θ*_0_,..., *θ*_*G *_depend on ***π ***and ***ρ ***as described in (2) and (3), and *C *is a constant independent of these parameters. Thus, for some choice of *K*, we estimate ***π ***and ***ρ ***by maximizing the criterion in (4), which only involves (*y*_1_,..., *y*_*G*_). This can be done with some iterative optimization algorithm. These estimates, denoted  and  for *k *= 1,..., *K*, are used in (3) and (2) to get the estimates of *θ*_0 _and this is in turn plugged into (1) to compute the corresponding prediction .

The final part of the estimation procedure is to find the proper number of components *K *in the binomial mixture, i.e how many binomial PMF do we need to approximate the distribution of the observed data (*y*_1_,..., *y*_*G*_). Since our criterion in (4) is a log-likelihood function for the data, we have adopted the Bayesian Information Criterion (BIC) to select the proper model complexity [9], a choice also supported by [10]. Hence, we look for a *K *where(5)

is minimized, where (2*K *- 2) is the number of free parameters in the model since the sum of mixing proportions is always 1.0 and the core component has a fixed detection probability *ρ*_1_.

Once we have determined the proper number of components *K *the estimated core- and pan-genome sizes are

where  is the estimated mixing proportion for component 1, the core component.

We have observed that the pan-size estimate may be heavily influenced by the chosen number of components, a generic property discussed by [10]. In order to stabilize the estimates, [10] propose a bagging-based estimator, which we have adopted. This is a bootstrap procedure that will smooth the estimate over various choices of components, and making the final estimate more stable.

As an alternative to the binomial mixture model estimate, we have also included the Chao lower-bound estimate [11] when fitting to real data. This is a very simple procedure, where the pan-genome size is estimated by

Notice that this corresponds to *y*_0 _being predicted from *y*_1 _and *y*_2 _only.

### Implementation

All computations, including the parsing of BLAST results, setting up the pan-matrix and performing all estimations have been implemented in R [12] and is freely available from the corresponding author. An R-package for microbial pan-genomics is under construction and will be made available as soon as it is operational.

### Testing

#### Estimating core- and pan-sizes

**Figure 2:**
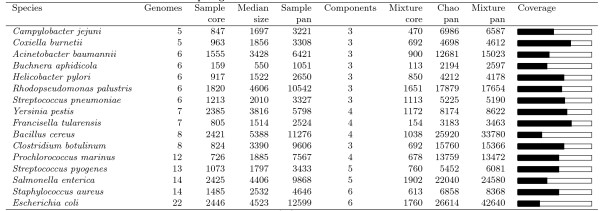
**Genomes and their core- and pan-genomes**. Number of genomes refer to completed genomes at NCBI [13] at the end of January 2009. Sample core, Median size and Sample pan are the observed quantities, while Mixture core, Chao pan and Mixture pan are estimated quantities. Components is the optimal choice of mixture components. The black bars under Coverage indicate pan-genome coverage, *i.e*. the current sample pan-genome size as a fraction of the estimated pan-genome size (Mixture pan).

We employed our method to data for 16 different bacterial species, who have all at least 5 different genomes sequenced and annotated at NCBI [13] on January 1. 2009. The gene families were computed, for each genome as described above. Estimated core- and pan-genome sizes are given in Figure 2. It is important to note that in this work we are discussing gene families, and not individual genes; although the two are closely related in bacteria, they are not identical. The number of components in the mixture-models was found by minimizing the BIC-criterion. The bars on the right-hand side of Figure 2 represent the fraction observed so far, of the total estimated pan-genome. *Francisella tularensis *currently has the largest fraction covered, at 73%; this seems reasonable, in that the total pan-genome for an intracellular organism would be expected to be relatively small, compared to environmental isolates. The bacterial species with the smallest fraction of the estimated total pan-genome covered is that of *E. coli*, with a mere 30% covered so far, based on 22 genomes completely sequenced.

**Figure 3:**
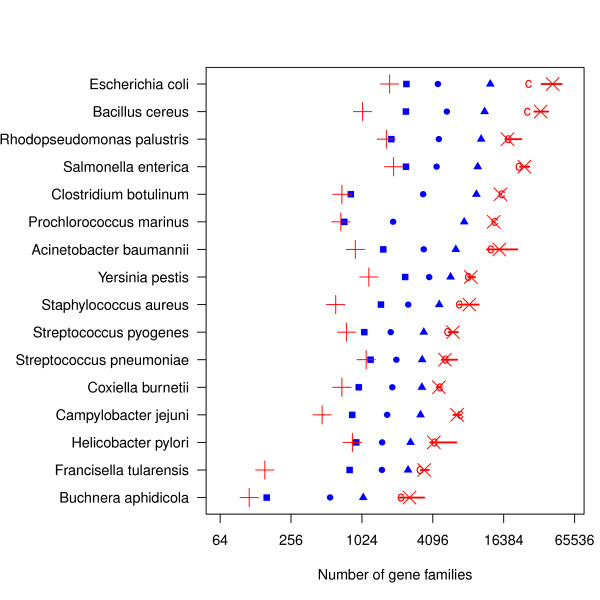
**Core- and pan-genome size estimates**. Observations and estimates of core- and pan-genome sizes. The horisontal axis is on log_2 _scale. Solid blue markers represent the observed data; squares are the core genes, circles are the median number of genes for an individual genome, and the triangles are the total number of gene families found in the data set. The red "+" represents the estimated core size, whilst the red "x" is the estimated size of the pan-genome using the binomial mixture model. The red "c" is the Chao lower-bound estimate of pan-size. The bars represents a 90% naive bootstrap confidence interval for the pan-genome, giving a rough indication of uncertainty.

Figure 3 shows estimates for the total number of gene families for the core- and pan-genomes of the 16 bacterial species. Note that for *E. coli*, the size of the estimated total pan-genome is about 43,000 gene families – or nearly twice the size of the human genome. On the other hand, for *B. aphidicola*, the total pan-genome is estimated to be about 2600 genes.

#### Distribution of gene families

**Figure 4:**
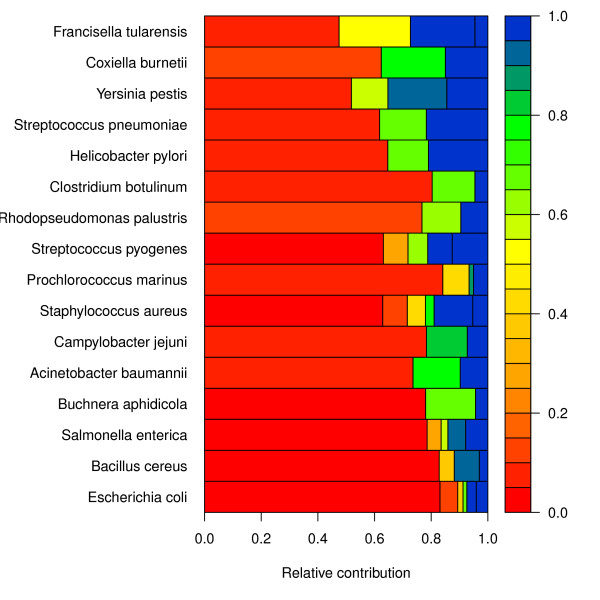
**Estimated mixture models**. Graphical display of binomial mixture models. Each rectangle corresponds to a component, its width indicates its mixing proportion and its color indicates its detection probability (see color bar). Red areas indicate parts of the pan-genome with a small detection probability, i.e. rarely occurring genes, whilst regions towards the blue end of the scale represent conserved genes – that is, genes shared by most of the genomes.

Figure 2 shows the coverage of the total pan-genome, for each species. In order to further explore the distribution of gene families within a species, and compare to other species, the mixture model components are informative. Figure 4 can be viewed as a graphical display of the binomial mixture models. Again, it is obvious from this figure that *E. coli *has only a fraction of the pan-genome covered by the observed data, with one quite large component that is red (very small detection probability). On the other hand, *F. tularensis *has most of the pan-genome already covered by the data examined; this is also the case for *Coxiella burnetii *and *Yersinia pestis*.

#### Effect of growing data set

**Figure 5:**
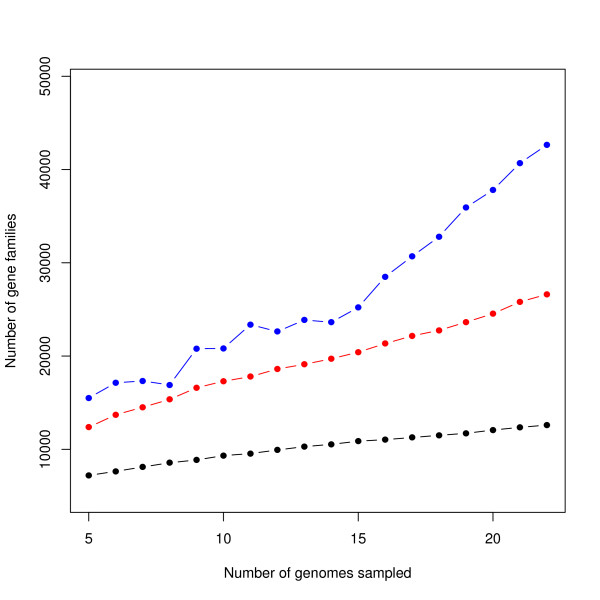
**Effect of growing E. coli data set**. Sample (black) and estimated population (red and blue) pan-genomes sizes for *E. coli*, as a function of number of genomes sampled. In blue is our mixture-model estimate, in red the Chao lower-bound estimate and the black is the observed size. All of these values are averages over 22 data sets. Note that for the lower number of genomes, the estimates tend to have larger variability, due to the larger number of ways to sample a small number of genomes out of a pool of 22 genomes; at the other end of the scale, the 22 possible combinations of 21 genomes are very similar to each other.

For one of the species, *E. coli*, we have already 22 fully sequenced genomes. Still, the coverage, defined as sample pan-genome size divided by estimated pan-genome size, is as low as 30%. An interesting question is of course how many more genomes do we need to sequence in order to have a coverage of, say, 90% of the *E. coli *pan-genome? Upon examination of this question, we discovered that this number appears to grow as more genomes are sequenced. That is, with only a few genomes sequenced, it might appear say that 100 genomes might be enough to cover the estimated pan-genome. However, even with only 22 genomes sequenced, now it looks as though perhaps around 220 additional *E. coli *genomes would be needed. In coming up with this estimate, we find that, as more *E. coli *genomes are sequenced, the total estimated diversity increases, resulting in a steep increase in the estimate of the pan-genome total size, as shown in Figure 5.

#### Effect of gene prediction

The use of a mixture model makes it apparent that the estimate of pan-genome size must depend on how many gene families we observe in few genomes. Especially those gene families observed in only one genome, are most likely important. These genes are often referred to as ORFans. Upon inspection of the data, we found that the annotation "hypothetical protein" is severely over-represented among the ORFans in all 16 species (Fisher exact test p-values less than 10^-10^). Thus, false positives from the gene prediction, *i.e*. predicted gene who are not actually genes, are most likely influencing the number of ORFans most since false positives typically are "hypothetical proteins". This makes the number of ORFans uncertain, and estimation of pan-genome size even more difficult.

In order to quantify this effect, we made a re-analysis of the *E. coli *data, which is the largest data set. First, we removed 10% and 50% of the shortest hypothetical proteins in the data set, because we believe these are the most uncertain predictions. A pan-genome size was estimated for these reduced data sets. Next, we also made a completely new prediction of genes for all 22 genomes using the Easygene tool ([14,15]), and made another estimate from these data as well. The results are displayed in Table 1. The number of ORFans drops dramatically consistent with the idea that perhaps a large fraction of the ORFans are due to artifacts of gene finding. The pan-size estimates also tend to decrease as an effect of this, but the mixture model estimates show some variability.

**Table 1:** Effect of gene predictions

Data set	Observed	ORFans	Chao	Bin. mix.
Original NCBI	12599	5438	26614	42640
Reduced 10%	11273	4470	22549	32528
Reduced 50%	9336	3272	17083	27456
Easygene	9211	3121	17041	29818

The number of observed gene families in data set, the number of ORFans (gene families found in 1 genome only), Chao estimates and binomial mixture estimates of pan-genome size for the original *E. coli *data as well as reduced data sets. "Reduced 10%" means the 10% shortest hypothetical proteins were removed from the original data set, and correspondingly for "Reduced 50%". "Easygene" is a new data set with genes predicted by the Easygene gene prediction tool.

## Discussion

The use of a binomial mixture model for estimating the pan-genome size was introduced by [7], but the use of mixture models for population size estimation is by no way new, *e.g*. [8,10,16]. The estimation of a population size has a long history in ecology, under the names of capture-recapture problems (*e.g*. [17]), or in epidemiology, called multiple record systems (*e.g*. [18]). Mixture models are suitable when we are faced with a larger number of recaptures/records/genomes and heterogeneous detection probabilities, which is exactly the case for pan-genomics.

From our results in Figure 2 we notice that for none of the species the optimal mixture model has 2 components. This would be expected if the gene pool could be divided into core-genes and dispensable genes, as implicitly assumed by [2,6]. There is always at least a third group, and frequently even more. This observation corresponds to the results shown by [19], where they find that for bacteria and archaea in general, genes could be divided into three classes; core (always occurring), shell (moderately occurring) and cloud (rarely occurring).

A reason for this heterogeneity in detection probabilities may be skewed sampling. If some of the sequenced genomes are sampled in the same "corner" of the population, the genes characteristic for this "corner" will occur more frequently than they should. Another reason may be that some genes are simply frequently occurring in the population, reflecting a divergence from a fairly recent ancestor. In this perspective, it must be expected that there is a large number of true detection probabilities, which is at least partly supported by the fact that the more genomes we consider the more components we estimate (see Figure 2).

The fact that microbial genomic diversity is caused by both vertical mutations and horizontal transfer makes it also plausible to expect heterogenous detection probabilities.

From Figure 2 we also see that even for 22 genomes (*E. coli*) we only estimate 6 components. In [7] a mixture of 7 components were used for a data set of 8 genomes, which seems to be a too complex model. Using too complex mixture models will tend to over-estimate the pan-genome size, since it makes the estimate of the smallest detection probability artificially small.

In Figure 3 we see that a larger sample pan-genome tends to result in a larger estimated pan-genome.

This is due to the fact that larger data sets allow more complex models, and more complex models allow more extreme estimates. Uncertainties, as indicated by the rough confidence intervals, also tend to grow when estimates grow, which is reasonable.

In Figure 4 we have constructed a way to plot the estimated mixture models for comparative pan-genomics. In this picture the actual size of the core- and pan-genome is not visible, but we focus instead on the relative distribution of detection probabilities. Some species, typically have a large proportion of stable genes (blue area), while at the other end of the scale we find those with little overlap between genomes. A larger number of components indicates a more complex pan-genome with respect to heterogeneity in detection probabilities.

From the results in Figure 3 we can compute the coverage for each species, which is simply the size of the sample pan-genome divided by the estimated pan-genome size. Ideally, we should expect this to increase as the number of genomes increase, because the sample pan size should approach the true pan size. There is no such tendency in our results. We even observe that two of the largest data sets (*S. enterica *and *E. coli*) have two of the smallest coverages. Figure 4 also clearly demonstrates that, at least for *E. coli*, as more genomes become available the pan-genome estimates get even higher. This is typical for a population with a large fraction of ORFans. Since ORFans have a small detection probability, only a few of them will show up in every genome. Hence, it requires a substantial number of genomes before we can estimate their true abundance. In this perspective, the binomial mixture model will tend to under-estimate the true pan-size for smaller data sets.

In Table 1 we show that there are effects of possible false positive predicted genes on the estimates of pan-genome size. By removing hypothetical proteins from the data set, the number of ORFans drops. This again leads to a decreased pan-size estimates. Predicting new genes with Easygene gives the largest reduction in ORFans, but the effect on the mixture model estimated pan-size is less. This is due to the fact that the mixture model depends on the entire data distribution, not only the ORFans.

Our approach assume a closed pan-genome, i.e. *η *is a parameter. In an open pan-genome, the total number of genes is not fixed, and in a very long term perspective this is most likely the case, assuming new genes form and old genes disappear. However, in a reasonably short time window, the number of genes available to any population must be limited, and can be assumed constant. Wether genes are shared vertically or horizontally within the population should have no impact on the closedness of the gene pool.

A recent publication [20] has suggested alternative ways of estimating pan-genome size, based on power-laws and regression. Our, more probabilistic approach, is fundamentally different, and more in line with existing methods in capture-recapture modelling. However, as suggested by the results in Table 1, a major problem in pan-genome size estimation is the fact that the data themselves are estimates, and thus the uncertainty in the computation of gene families will influence the results, sometimes severely. In order to improve the estimation of bacterial genomic diversity, future efforts should probably be focused on this aspect.

## Conclusion

We have shown how to use binomial mixture models to estimate microbial core- and pan-genome size, and the vast literature on capture-recapture methods should be further exploited in microbial pangenomics, as it has been in closely related fields like metagenomics [21]. Our results indicate that pan-genomes for bacterial species are in general large compared to the size of individual genomes. Especially for *E. coli*, who has the largest number of completely sequenced and annotated genomes so far, we find that the pan-genome is significantly larger than the human genome. We also show that our pan-size estimates are most likely too moderate since the addition of new genomes tend to push them upwards. In order to improve reliability of estimates, more focus should be devoted to the computation of gene families.

## Authors' contributions

LS launched the idea of using capture-recapture methods and has done all programming and data analysis. TA has contributed to the choices of statistical methods and how to present them to a broader audience. DWU formulated the problem and supervised the choice of analyses to conduct. LS and DWU drafted the manuscript. All authors have read and approved the final manuscript.
